# Improvement in Acid Resistance of Polyimide Membranes: A Sustainable Cross-Linking Approach via Green-Solvent-Based Fenton Reaction

**DOI:** 10.3390/polym15020264

**Published:** 2023-01-04

**Authors:** Srinath Ravi, Woo-Seok Kang, Hyung-Kae Lee, You-In Park, Hosik Park, In-Chul Kim, Young-Nam Kwon

**Affiliations:** 1 School of Urban and Environmental Engineering, Ulsan National Institute of Science and Technology (UNIST), Ulsan 44919, Republic of Korea; 2 Membrane Research Center, Korea Research Institute of Chemical Technology, Daejeon 34114, Republic of Korea

**Keywords:** polyimide, Fenton reaction, acid resistance, nanofiltration membrane

## Abstract

In this study, we present a facile surface modification method using green solvents for a commercial polyimide (PI) nanofiltration membrane to exhibit good acid stability. To enhance acid stability, the PI organic solvent nanofiltration membrane was modified using Fenton’s reaction, an oxidative cross-linking process, using environmentally friendly solvents: water and ethanol. The surface properties of the pristine and modified PI membranes were investigated and compared using various analytical tools. We studied the surface morphology using scanning electron microscopy, performed elemental analysis using X-ray photoelectron spectroscopy, investigated chemical bonds using attenuated total reflectance-Fourier transform infrared spectroscopy, and studied thermal stability using thermogravimetric analysis. The acid resistances of the pristine and modified membranes were confirmed through performance tests. The pristine PI nanofiltration membrane exposed to a 50 *w*/*v*% sulfuric acid for 4 h showed an increase in the normalized water flux to 205% and a decrease in the MgSO_4_ normalized rejection to 44%, revealing damage to the membrane. The membrane modified by the Fenton reaction exhibited a decline in flux and improved rejection, which are typical performance changes after surface modification. However, the Fenton-modified membrane exposed to 50 *w*/*v*% sulfuric acid for 4 h showed a flux increase of 7% and a rejection increase of 4%, indicating improved acid resistance. Furthermore, the Fenton post-treatment enhanced the thermal stability and organic solvent resistance of the PI membrane. This study shows that the acid resistance of PI membranes can be successfully improved by a novel and facile Fenton reaction using green solvents.

## 1. Introduction

Water is vital for living organisms and is a crucial resource for the sustainable development of the earth’s environment. Today, approximately 88% of developing countries face water shortages and scarcity [1]. Therefore, state-of-the-art water and wastewater treatment technologies are required to overcome this problem. Membrane separation processes are widely used in water and wastewater treatment. Among the various pressure-driven membranes, nanofiltration (NF) membranes, which have a pore diameter of approximately 0.5–2.0 nm, have recently attracted significant attention [2]. This is mainly attributed to their lower required pressure, high permeate flux, cost-effectiveness, high retention of multivalent salts, and ability to separate low molecular weight organic compounds (i.e., those with molecular weight in the range 200–1000 g mol^−1^)) from solutions containing monovalent ions. Recently, there has been an increasing demand for acid-resistant NF membranes, particularly for the recovery of target chemical compounds from highly acidic aqueous and organic solutions. NF membranes are used particularly widely in the pulp and paper industries for the treatment of acidic effluents [3], the mining and metal industries for the removal of heavy metals [4] and sulfate ions [5], dairy cleaning-in-place processes for the regeneration of acid streams [6], gold ore processing for the removal of gold from solutions with high sulfuric acid concentrations [4], and sewage sludge treatment for the recovery of phosphorous [7,8]. Other uses of acid-resistant NF membranes include the separation of rare-earth elements, such as lanthanum and yttrium, from strong acids, such as nitric acid (HNO_3_), hydrochloric acid (HCl), and sulfuric acid (H_2_SO_4_) [9]. NF membranes are also used in the semiconductor industry for the treatment of the etching wastewater containing HCl, hydrobromic acid (HBr), and hydroiodic acid (HI) [10,11]. In all the aforementioned applications, membranes must operate in low pH solutions. Although high-performance commercial NF full-aromatic polyamide membranes, which are fabricated from piperazine (PIP) or m-phenylenediamine (MPD) with trimesoyl chloride (TMC), are available, their application is limited to the pH range of 2–11 [11,12,13].

Among a wide range of polymer materials, polyimide (PI) is known for its good chemical, thermal, and mechanical stability, as well as exceptional engineering processability [14,15]. Modified PI show enhanced chemical stability, that is, excellent resistance to many polar aprotic organic solvents, because of the rigid structure of the polymer backbone [16,17]. Diamines are the most commonly used crosslinkers for modifying PI membranes. Diamines react with a carboxylic group in the form of poly(amic acid) to form a bridge between two polymer chains. Modification with diamines is performed by adding them to the polymer casting solution. Zhao et al. [18] added a cross-linking agent to a polymer solution with a concentration of less than 5 wt.% to avoid gel formation. The modified PI membrane was more flexible than the unmodified PI membrane and demonstrated higher gas permeability and selectivity. This method of adding a cross-linking agent to the casting solution can only be used with polymer solutions of very low concentration to avoid gelation of the casting solution before film synthesis. Hence, its use is limited to the production of thin and dense PI films suitable for gas separation. At the same time, there are cases in which cross-linking is performed via post-treatment of the fabricated membrane. Toh et al. [19] modified Lenzing P84 PI membranes with aliphatic diamines (ethylenediamine (EDA), propylenediamine, 1,6-hexanediamine, and 1,8-octanediamine). The modified membranes showed excellent resistance to N,N-dimethylacetamide (DMA), dimethylsulfoxide (DMSO), N-methylpyrrolidone (NMP), and N,N’-dimethylformamide (DMF); furthermore, using the filtration test with toluene, they elucidated that cross-linking reduces the flux by making the active layer denser. The membrane modified using EDA showed a high flux in DMF; moreover, its stable performance was confirmed even after 120 h. Vanherck et al. [20] modified Matrimid PI membranes with aromatic diamine p-xylenediamine to prepare stable membranes for the filtration of DMA, DMSO, NMP, tetrahydrofuran, and DMF. With increasing cross-linking time, the flux decreased, indicating that the active layer became denser. The membrane showed excellent performance in DMF, a flux of up to 5 LMH, and the rejection of methyl orange of up to 98%. In the development of membranes for the treatment of solutions containing both organic solvents and acids, it may be a promising strategy to impart acid resistance to organic-solvent-resistant PI membranes via post-treatment. 

**Figure 1:**
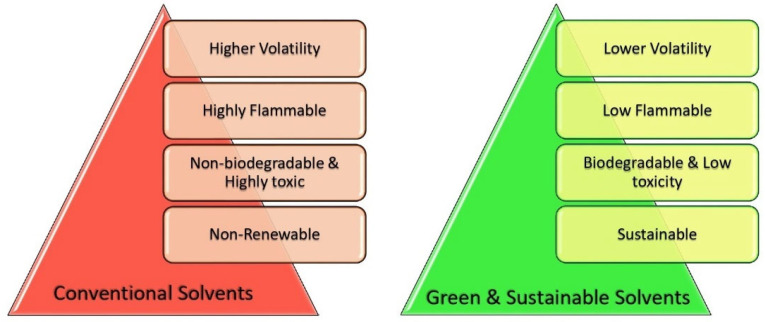
Merits of green and sustainable solvents over conventional solvents.

Recently, sustainability and green chemistry have attracted considerable interest for reducing the worldwide usage of toxic chemicals and pollution. However, in most membrane fabrication and modification processes, conventional toxic solvents are used [21,22]; therefore, studies have been currently focused on replacing these solvents with non-toxic or green alternatives. Figure 1 presents the major merits of using green solvents as replacements for toxic solvents. The expression “green chemistry” was introduced to attract the attention of scientists worldwide to limit the utilization of hazardous and toxic reagents or solvents in the production of chemicals [23]. 

Zhao et al. cross-linked the polybenzimidazole (PBI) backbone using potassium persulfate as an oxidant; this method is categorized as “green” because of the use of an aqueous solution instead of toxic solvents [24]. In another study, Shin et al. modified a PBI organic-solvent-resistant NF membrane using oxidative cross-linking with an aqueous solution of potassium permanganate (KMnO_4_) [25]. Membrane science and technology is one of the crucial components of green chemistry because it plays a major role in the environmental friendliness of industrial processes. Although recent advancements in the field of membrane fabrication and performance enhancement are commendable, the materials used must be revised to reduce the pollution and pernicious effects of toxic chemicals [26,27,28]. 

Herein, a novel facile method for cross-linking the commercially available PI NF membranes via post-treatment is introduced to improve their acid stability. The Fenton reaction, an oxidative cross-linking process involving catalytic decomposition of hydrogen peroxide (H_2_O_2_) to hydroxyl radicals in presence of ferrous iron, was applied to crosslink and improve the acid resistance of the membrane. In this method, green solvents (water and ethanol) are used, making it a sustainable approach to cross-linking/modification of PI membranes. The surface properties of the pristine and modified PI membranes were studied using various characterization methods; the acid stability of the membranes was investigated using 50 *w*/*v*% H_2_SO_4_, and the water flux and MgSO_4_ rejection were determined through a filtration test. Changes in the surface properties of the PI membranes after exposure to acid were evaluated using various analytical tools. 

## 2. Materials and Methods

### 2.1. Materials

DuraMem^®^900 (Flat sheet P84 PI membrane, Evonik (Essen, Germany)), a commercially available PI membrane, was used as a PI NF membrane, the acid stability of which was improved. Milli-Q water, ethyl alcohol (Daejung (Siheung, Republic of Korea), 95%), iron (II) sulfate heptahydrate (Sigma-Aldrich (St. Louis, MO, USA), 99%), and H_2_O_2_ (Daejung (Siheung, Republic of Korea), 30%) were used for the Fenton reaction. MPD (Sigma-Aldrich (St. Louis, MO, USA), 99%) was used as an additive in the Fenton reaction. H_2_SO_4_ (Daejung (Siheung, Republic of Korea), 95%) was used in the acid stability test, and MgSO_4_ (Daejung (Siheung, Republic of Korea), 99%) was used to prepare the conductive feed solution. All chemicals were used as received. 

### 2.2. Modification of PI Membrane via Fenton Reaction

Prior to modification, the PI membrane was immersed in running deionized (DI) water for 24 h to remove any residual chemicals present on its surface. Iron sulfate (2.8 g), ethanol (100 g), and Milli-Q water (100 g) were mixed in a 250 mL glass screw-cap reagent bottle. The mixture was continuously magnetically stirred at 50 °C. The pristine PI membrane was immersed in the reaction mixture and purged with nitrogen. Under a nitrogen atmosphere, 12 g of H_2_O_2_ was added to the reaction mixture and allowed to react for 1 h. After the reaction completion, the membrane was thoroughly washed with running DI water and stored in Milli-Q water for further characterization and performance tests.

The effect of the MPD additive was evaluated during the Fenton reaction. In a 250 mL glass screw-cap reagent bottle, aqueous solutions (100 g) of MPD with various wt.% were prepared by dissolving 0, 0.2, 1.5, and 2.5 g of MPD in Milli-Q water. The mixture was stirred thoroughly to obtain a homogeneous solution. To each of these 100 g aqueous MPD solutions, 2.8 g of iron sulfate and 100 g of ethanol were added and stirred. Thereafter, the Fenton reaction was conducted as described above. 

### 2.3. Filtration Performance of the Membrane

The performance of the pristine and modified PI membranes was evaluated using filtration test equipment similar to that used in other studies [29]. The filtration test was performed at 25 °C at a flow rate of 2.5 L/min using 2000 ppm MgSO_4_ aqueous solution. Four membrane samples with an active layer area of 19.6 cm^2^ were installed and processed simultaneously in the filtration system. Before sampling, the filtration test procedure was followed by compaction at 150 psi for 1 h and stabilization at 75 psi for 30 min. Water flux and salt rejection were measured three times, each for 10 min at 75 psi. Water flux was calculated as the change in volume of the permeate solution (converted from weight change and density of the solution) per unit membrane area and time, as in Equation (1). The concentrations of the feed and permeate solutions were calculated using a conductivity meter, and the salt rejection of MgSO_4_ was determined according to Equation (2).

(1) $$J=\frac{△w}{t\times A\times \rho }$$

*J* = water flux (L/m^2^h)
*w* = weight of permeate water (g)
*t* = time (h)
*A* = effective membrane area (m^2^)
*ρ* = density of water (g/L)

(2) $$R\left(\%\right)=\left(1-\frac{C_{permeate}}{C_{feed}}\right)\times 100$$

### 2.4. Acid Resistance and Organic Solvent Stability Test

The acid resistance of the pristine and modified membranes was evaluated by exposing them to 50 *w*/*v*% H_2_SO_4_ at room temperature for 4 h. The acid-exposed membrane was thoroughly washed with DI water, and a performance test was conducted as described in Section 2.3. Unless indicated otherwise, acid-resistance tests were performed under these conditions. However, for the long-term exposure test, the membrane was exposed to 15 *w*/*v*% H_2_SO_4_ for 240 h. J

The organic solvent stability of the PI membranes was determined by comparing the weights of the membranes before and after exposure to the organic solvent of interest for a specified time. Membrane samples were washed with DI water and completely dried in an oven at 100 °C. The active layer was separated from the support layer to exclude the effect of the support layer during the solvent exposure test. It was weighed and exposed to dimethylacetamide (DMAc) in a glass vial at 80 °C for 20 h. After 20 h of exposure, the residual active layer was washed and then dried for at least 24 h in an oven at 100 °C. The ratio of the weight before exposure to that after exposure was calculated using Equation (3). The solvent resistance of the membrane samples was then compared.

(3) $$W\left(\%\right)=\frac{Weight\;after\;solvent\;exposure\;}{Weight\;before\;solvent\;exposure}\times 100$$

### 2.5. Characterization

#### 2.5.1. Scanning Electron Microscopy (SEM) and Attenuated Total Reflectance-Fourier Transform Infrared Spectroscopy (ATR-FTIR)

SEM (S-4800, Hitachi High-Technology (Tokyo, Japan)) was used to investigate the surface morphology of the membrane before and after exposure to acid and before and after the modification of the membrane. The analyzed membrane was immersed in DI water and freeze-dried for at least 72 h prior to analysis. The freeze-dried membrane was coated with Pt at 20 mA and 2 × 10^−3^ mbar for 60 s in a turbo-pumped high-resolution chromium sputter coater (K575X, EMITECH, Lohmar, Germany) to reduce image artifacts caused by static electricity. 

ATR-FTIR (Nicolet 6700, Thermo Fisher, Waltham, MA, USA) was used to investigate the chemical properties of the membranes. The freeze-dried membrane samples were analyzed using Ge crystals. ATR-FTIR was conducted at a resolution of 4 cm^−1^ in the range 700–3700 cm^−1^, and the obtained spectra were analyzed using Omnic 8.1 software.

#### 2.5.2. X-ray Photoelectron Spectroscopy (XPS) and Thermo-Gravimetric Analysis (TGA)

XPS was used to evaluate the effect of the Fenton reaction on the elemental composition of PI membranes. XPS spectra were obtained in the range of the electron binding energy of 0–1000 eV with a resolution of 1 eV. In this study, the binding energies of C1s, O1s, and N1s were detected at 285, 531, and 399 eV, respectively.

TGA (Q500, TA Instruments (New Castle, DE, USA) was used to investigate the thermal stability of the PI membranes. The membrane sample immersed in DI water was cut into 2 cm × 2 cm pieces and freeze-dried for at least 72 h. The test was performed at 30–800 °C and a ramp rate of 10 °C/min. The effect of the Fenton reaction on thermal stability was investigated based on the difference in weight between the pristine and modified membranes. 

#### 2.5.3. Molecular Weight Cut-Off (MWCO) and Pore Size of Membranes

MWCO of the membranes was determined by the molecular weight of the neutral solute, in which 90% of the solute was retained by the membrane. The MWCO values of the pristine and Fenton-modified membranes were obtained using the previously reported method [30,31,32]. Filtration tests were conducted using the feed solutions of 50 ppm polyethylene glycol (PEG) with molecular weights of 400, 600, 1000, and 2000 Da as a model solute at 25 °C and a flow rate of 2.5 L/min. The concentrations of PEGs in the feed and permeate were measured using a total organic carbon analyzer (TOC-V, Shimadzu, Japan). Because the molecular weight (*M_w_*, Da) of the solute, PEG, is related to the Stokes–Einstein radius (*R*, cm), the average pore size of the membrane can be estimated [33] using

(4) $$R=\left(16.73\times 10^{-10}\right)M_{w}^{0.557}$$

## 3. Result and Discussion 

### 3.1. Performance Degradation of the PI Membrane Owing to Acid Exposure

**Figure 2:**
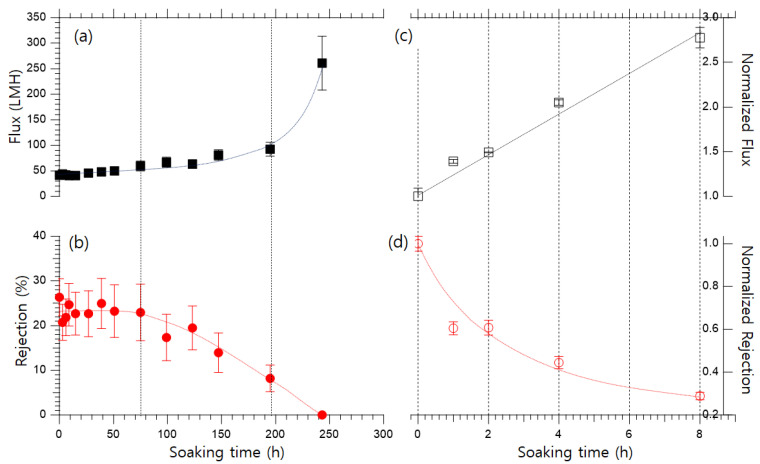
(**a**) Flux and (**b**) MgSO_4_ rejection of the PI membrane after long-term exposure to 15 *w*/*v*% H_2_SO_4_. (**c**) Normalized flux and (**d**) normalized rejection of the PI membrane after exposure to 50 *w*/*v*% H_2_SO_4_.

The acid resistance of PI membranes was evaluated by exposure to 15 *w*/*v*% H_2_SO_4_ for 240 h. The pH values of the H_2_SO_4_ solutions with pKa of −3 and 1.9 [34] are approximately −0.2. The flux and rejection of the PI membrane after exposure to 15 *w*/*v*% H_2_SO_4_ (Figure 2a and 2b, respectively) can be categorized into three phases. In phase I, the flux slightly increases, and the rejection remains almost constant. In phase II, the flux gradually increases, but rejection monotonically decreases. In phase III, the flux rapidly increases, and the membrane loses its capability to reject ions from the feed solution. From these results, it can be inferred that the PI membrane has a certain tolerance for acid, but beyond this range, the membrane degrades under acidic conditions. 

To ensure that the experiment is systematic and time-efficient, the concentration of H_2_SO_4_ was increased to 50 *w*/*v*%. The pH of 50 *w*/*v*% H_2_SO_4_ was −0.7~−0.8. The performance of the acid-exposed membrane was expressed as the normalized flux and normalized rejection and compared to the performance of the pristine membrane. The normalized flux after exposure to 50 *w*/*v*% H_2_SO_4_ for various time intervals is shown in Figure 2c. The flux of the PI membrane after exposure to the acidic solution for 1, 2, 4, or 8 h increased to 139, 149, 205, and 278%, respectively, and the normalized rejection, as shown in Figure 2d, decreased to 60, 60, 44, and 28%, respectively. Thus, the damage to the PI membrane is strongly dependent on the exposure time. Exposure to 50 *w*/*v*% H_2_SO_4_ for 4 h was used as the standard exposure condition in further experiments. 

### 3.2. Fenton Reaction to Improve acid Resistance of PI Membranes

**Figure 3:**
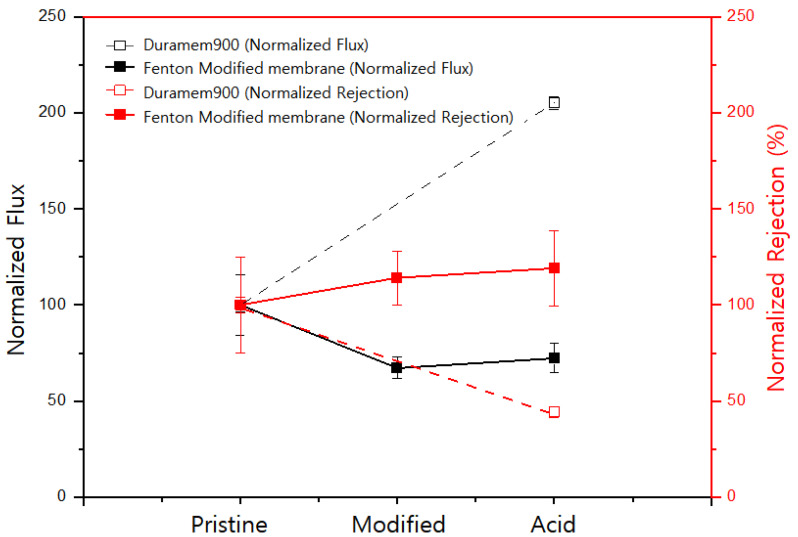
Normalized flux and normalized rejection of pristine and Fenton-modified PI membranes. The membrane was modified by the Fenton reaction for 1 h.

**Figure 4:**
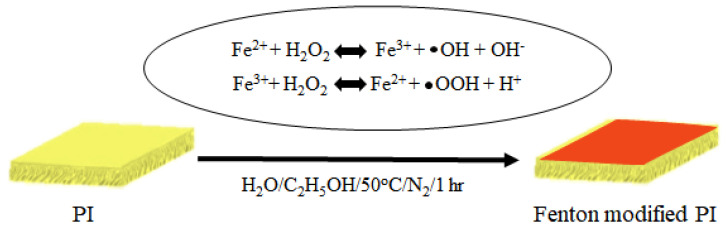
Schematic of Fenton modification of the PI membrane.

The performance of pristine, modified, and acid-exposed membranes is shown in Figure 3. After exposure to 50 *w*/*v*% H_2_SO_4_, the normalized flux increased to 205%, and the normalized rejection decreased to 44% (dashed line in Figure 3), indicating that the PI membrane was damaged by acid exposure. When ferrous ions (Fe^2+^) are added to H_2_O_2_ solution, ferrous ions are oxidized to ferric ions (Fe^3+^), and H_2_O_2_ is converted into hydroxyl free radicals and hydroxyl anions. Then, the ferric ions are again reduced to ferrous ions, generating protons and hydroperoxyl free radicals from other H_2_O_2_ molecules (Figure 4) [35,36]. Iron ions serve as catalysts for the generation of free radicals during the reaction. The generated free radicals reacted with the PI to lower the flux and slightly increase the rejection. This is likely due to the cross-linking of the membrane. More details on the reaction mechanism are provided in the analysis of ATR-FTIR spectra.

**Figure 5:**
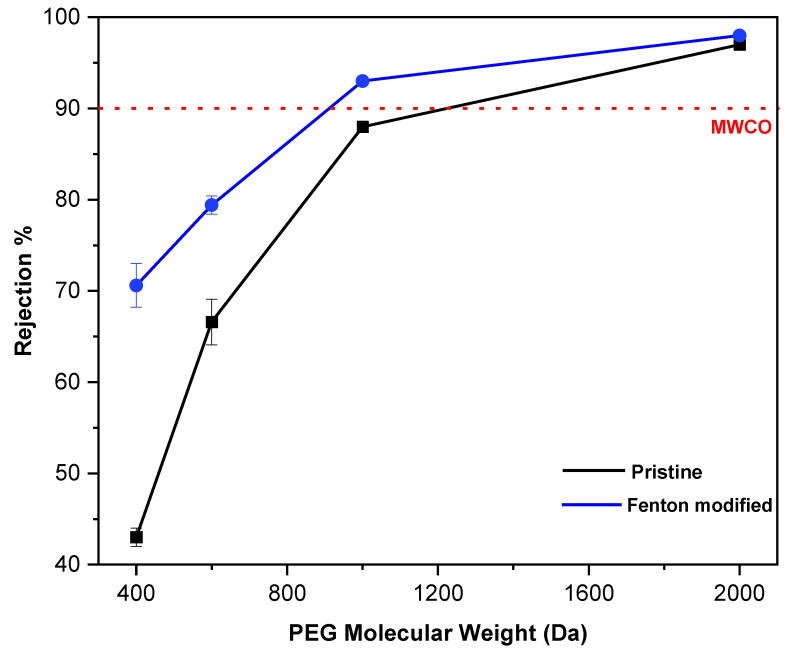
PEGs rejection curves of pristine and Fenton-modified PI membranes for MWCO measurement.

The change in the pore size of the membrane by the Fenton reaction was evaluated through the rejection of a neutral solute passing through the membrane. The molecular weight of the solute at which the rejection reaches 90% is called MWCO. Figure 5 shows the rejection of PEG solutes with various molecular weights for both pristine and Fenton-modified PI membranes. The rejection of both membranes increases with increasing molecular weight of the PEG solute from 400 to 1200 Da. The MWCO of the pristine PI membrane was ~1060 Da, whereas that of the Fenton post-treated PI membrane was ~920 Da. These values correspond to the mean pore size of ~0.81 and ~0.74 nm, respectively. The reduction of pore size by the Fenton reaction explains the decrease in flux and a slight increase in the rejection, as shown in Figure 3.

At the same time, exposure of the Fenton-modified membrane to 50 *w*/*v*% H_2_SO_4_ resulted in 7% and 4% increases in normalized flux and rejection, respectively. Compared with the pristine membrane, the modified membrane showed little change in performance after exposure to H_2_SO_4_. Thus, Fenton modification enhanced the acid resistance of the membrane. 

**Figure 6:**
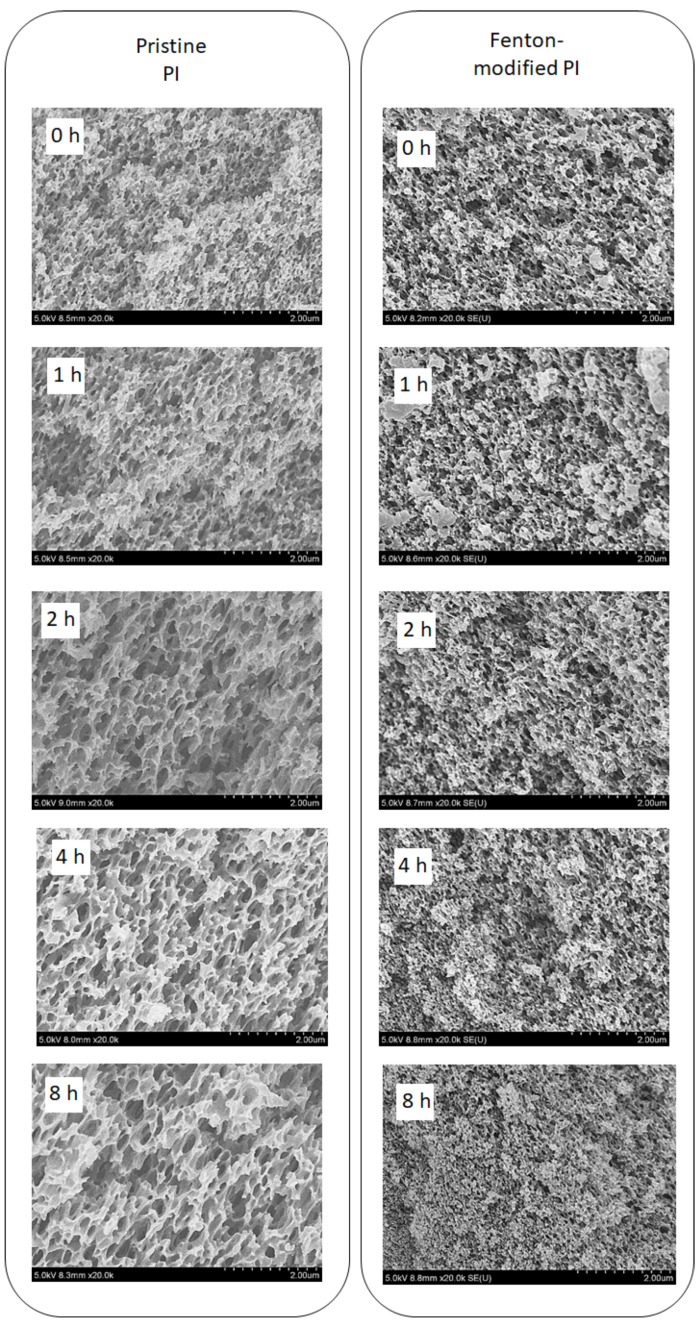
SEM images of pristine and Fenton-modified PI membranes after exposure to 50 *w*/*v*% H_2_SO_4_ for 0, 1, 2, 4, and 8 h.

The structure of PI membranes was characterized using SEM. Cross-sectional SEM images of pristine and Fenton-modified PI membranes after exposure to 50 *w*/*v*% H_2_SO_4_ for 0, 1, 2, 4, and 8 h are shown in Figure 6. As the exposure time to the H_2_SO_4_ solution increases, the pore size of the pristine PI membrane also increases. This indicates that harsh acidic conditions led to polymer degradation. This result is consistent with the deterioration of the membrane performance shown by the increase in flux and decrease in the rejection upon exposure to acid (Figure 2). However, the pore size of the Fenton-modified membrane did not increase with the exposure time to the H_2_SO_4_ solution. Therefore, the Fenton-modified membrane has superior acid resistance, which is attributed to the cross-linking of the PI membrane by the Fenton modification. 

**Figure 7:**
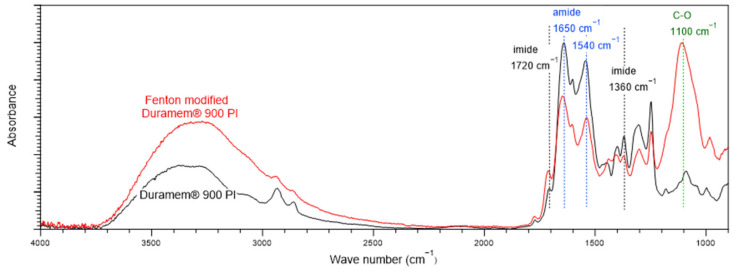
ATR-FTIR spectra of pristine and Fenton-modified PI membranes.

**Figure 8:**
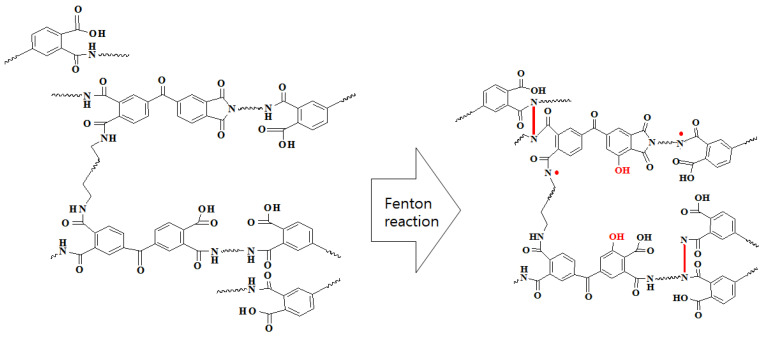
Cross-linking of Duramem900 PI membrane using the Fenton reaction.

Figure 7 shows the FTIR spectra of the DuraMem^®^900 PI membranes. Symmetric C=O stretching and C–N–C stretching of the imide peak are observed at 1720 cm^−1^ and at 1360 cm^−1^, respectively [19,20]. The peak of O–H stretching is distributed at 2500–3300 cm^−1^ [37], and the peaks of the CONH structure of the amide group, known as amide I and amide II bands, are observed at 1650 and 1540 cm^−1^, respectively [21]. The amide I band was mainly attributed to C=O stretching, and the amide II band was ascribed to the combination of N–H in-plane bending and N–C stretching of the amide bond [38]. Both imide and imide functional groups were also detected in the pristine PI membranes. In the Fenton-modified membrane, the peak of O–H stretching located between 3300 and 2500 cm^−1^ is more pronounced, and the CO peak of the secondary alcohol at 1100 cm^−1^ [39,40] is significantly more intense than in the pristine membrane. The PI membrane used in this study was a DuraMem^®^900 PI membrane (Evonik), which is a commercially available organic-solvent-resistant membrane. In the post-treatment for imparting organic solvent resistance to PI membranes, imide functional groups are commonly converted into amide and carboxylic groups [19]. When PI is immersed in an alkaline solution, the imide ring can be broken to form polyamic acid, which can react with other amines to form polyamide functional group [41]. According to ATR-FTIR, the organic-solvent-resistant PI membrane contains both amide and imide groups. It has been reported that amide bonds are vulnerable to acids [12,29]. The hydrolysis of amides begins when (i) the proton attacks the oxygen or nitrogen of the amide bond, followed by (ii) nucleophilic addition of water molecules to form a dihydroxy tetrahedral intermediate structure, (iii) transfer of the hydroxyl group from the oxygen to the nitrogen of the amide bond, and (iv) breakage of the amide bond [13,30]. During Fenton post-treatment of the membrane, the amine of the amide functional group can be attacked by hydroxyl radicals produced by Fenton reagents, leading to the radicalization of the amine. The radicalized amines react with each other to form a cross-linking bond (Figure 8). The cross-linking of the membrane might enhance the acid resistance of the membrane. The degree of stability of the amide bond is affected by the resonance between the electrons of the C=O π-bond and lone pair electrons of nitrogen [14]. When the nitrogen atoms of the amide bonds are bonded to each other (–CO–$\ddot{N}$–$\ddot{N}$–O–) by Fenton post-treatment, the resonance structures of the two amide bonds overlap and expand, thereby strengthening the resonance structure and enhancing the acid tolerance of the membrane. 

The surface chemistry changes in the PI membrane owing to the Fenton post-treatment were evaluated using XPS, and the results are shown in Table 1. The elemental composition of the pristine membrane was 73.0% carbon, 10.7% nitrogen, and 16.3% oxygen. However, in the Fenton-modified membrane, the atomic percentage of oxygen is significantly higher (58.3%). This result can be explained by the increase in the contents of hydroxyl and carboxylic functional groups generated in the PI membrane during the oxidation by H_2_O_2_ [42,43], as shown in the ATR-FTIR spectra (Figure 7). The decrease in the atomic percentages of carbon and nitrogen is attributed to the decrease in the total carbon and nitrogen content owing to the significantly increased oxygen content. 

**Figure 9:**
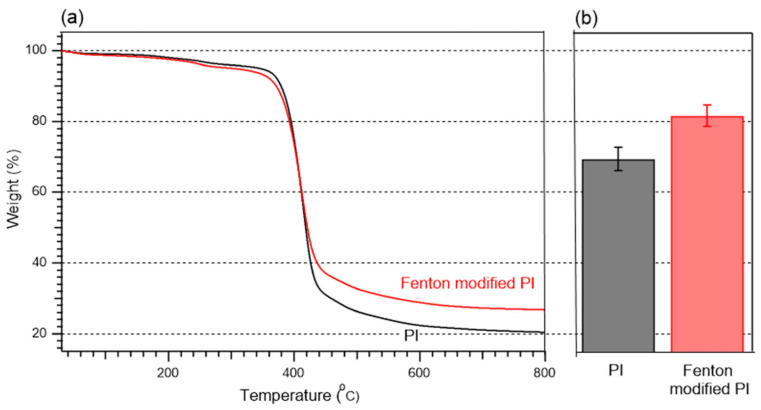
(**a**) TGA spectrum of pristine and modified membranes and (**b**) residual weight of pristine and Fenton-modified membranes soaked in DMAC at 80 °C for 20 h.

Figure 9 shows the TGA results for the membrane samples used in this study. Both samples show a rapid mass loss at ~400 °C, which was attributed to the degradation of the imide group [44,45]. At 800 °C, the residual masses of the pristine and Fenton-modified membranes were 20.3 and 26.7%, respectively. The Fenton-modified membrane shows better thermal durability, suggesting that cross-linking was realized through the Fenton reaction. The effect of the cross-linking of the PI membrane can also be observed by its solubility in polar aprotic solvents such as DMAc. Figure 9b shows the residual weight of the pristine and Fenton-modified membranes after the solvent exposure test. The pristine PI membrane retained 69.4% of its original weight after soaking at 80 °C for 20 h, whereas the Fenton-modified membrane retained 81.6%, demonstrating successful cross-linking and enhanced solvent resistance compared to that of the pristine membrane. Thus, Fenton post-treatment not only increased the acid resistance but also further improved the thermal stability and organic solvent resistance.

Table 2 presents a comparison of the solvents used for cross-linking the PI membrane and the performance of the membrane. Compared to other studies, the Fenton method requires less time for modification (1 h) and uses green solvents and non-toxic chemicals. The Fenton reaction of DuraMem^®^ PI is a facile and sustainable process to enhance the acid resistance of PI membranes for the treatment of solutions containing organic solvents and strong acids.

### 3.3. Effect of MPD Additive in the Fenton Reaction 

PI membranes have been applied in various separation processes owing to their excellent chemical resistance. In certain cases, depending on the material to be separated from the feed solution, the rejection of commercially available membranes needs to be tailored. In particular, in pharmaceutical processes using both strong acids and organic solvents, the recovery of specific substances is important. A simple method for obtaining the desired performance by controlling the rejection (or recovery) via the addition of MPD during Fenton modification is proposed.

**Figure 10:**
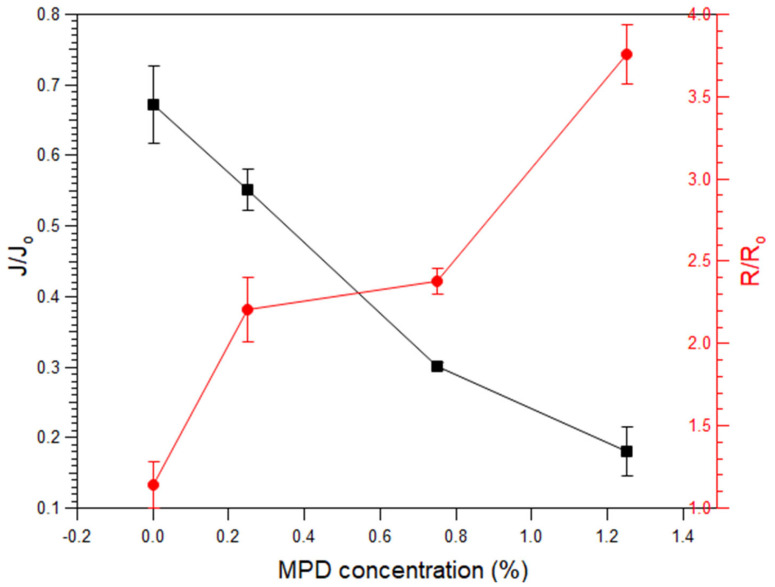
*J/J*_0_ and *R/R*_0_ values of Fenton-MPD-modified membranes.

The performance of the PI membrane modified by the Fenton reaction in the presence of MPD is shown in Figure 10. The concentrations of MPD added were 0, 0.25, 0.75, and 1.25%. When the concentration was increased to 2.25 and 3.5%, the flux significantly decreased to 0.8 and 0.5%, respectively, and the amount of permeate was insufficient for the rejection calculations. After the Fenton reaction, the *J/J*_0_ value was calculated to easily determine the change in the filtration characteristics of the membrane. *J*_0_ is the initial flux and *J* is the flux after modification. The *J/J*_0_ values of 0.67, 0.55, 0.30, and 0.18 were obtained at 0, 0.25, 0.75, and 1.25% MPD concentrations, respectively. An increase in the concentration of MPD resulted in a decrease in the flux after modification. Rejection was assessed by *R/R*_0_, which was calculated in the same way as the flux. The initial rejection is indicated by *R*_0_, and rejection after modification is indicated by *R*. *R/R*_0_ values of 1.14, 2.21, 2.37, and 3.76 are observed at MPD concentrations of 0, 0.25, 0.75, and 1.25%, respectively. An *R/R*_0_ value greater than 1 indicates that the rejection was increased by modification. The addition of MPD during the Fenton reaction increased the cross-linking of the polymeric membranes, thereby reducing the permeability and increasing the removal rate. However, the addition of MPD did not further increase acid resistance. According to the permeation test results, the performance of the acid-resistant and organic solvent-resistant DuraMem^®^ PI membranes can be controlled by the addition of MPD, leading to the potential extension of the application scope.

## 4. Conclusions

In this study, the DuraMem^®^ 900 PI membrane was modified using the strong oxidizing agent generated by the green solvent-based Fenton reaction. Pristine PI membrane exhibited a flux increase to 205% and a decrease in rejection to 44% after exposure to 50 *w*/*v*% H_2_SO_4_ at 25 °C for 4 h, which was used as the evaluation standard for acid resistance. These changes resulted from the degradation of the PI membrane in low pH environment. Fenton post-treatment induced cross-linking of the PI membrane, thereby reducing the flux and increasing the rejection. However, exposure of the Fenton-modified membrane to H_2_SO_4_ led to only a slight increase in flux and almost no changes in rejection. The modification through the Fenton reaction significantly improved the acid resistance of the PI membrane. The Fenton post-treatment of the PI membrane not only increased the acid resistance but also improved its thermal stability and organic solvent resistance. Furthermore, the performance of the acid-resistant and organic-solvent-resistant PI membranes was controlled using the MPD additive, thereby extending the application scope of the PI membrane modified via Fenton post-treatment.

## Figures and Tables

**Table 1:** Elemental compositions of pristine and Fenton cross-linked membranes according to XPS.

	Pristine	Fenton
Carbon (%)	73.0	36.2
Nitrogen (%)	10.7	5.5
Oxygen (%)	16.3	58.3

**Table 2:** Comparison of crosslinking methods of PI membranes.

Membranes	Crosslinking Method	Solvents Used for Crosslinking	Reaction Time Used for Crosslinking	Solute	Rejection (%)	Application	Reference
DuraMem^®^900 PI	Fenton reaction (oxidative crosslinking—Iron sulfate + Hydrogens peroxide)	Ethanol and Water	1 h	PEG 1000	~93	Acid Resistant Membrane (ARM)	This work
Synthesized PI (6FDA + Durene + DABA)	Cu^2+^ crosslinking between carboxylic acid of synthesized PI	Methanol	24 h	CBB	~99%	OSN	[46]
Lenzing P84 PI	Crosslinking of PI with diamine (HDA)	Isopropyl alcohol	24 h	RDB (479) RB (1017)	9899.9	OSN	[17]
Matrimid^®^ 5218 PI	Imide ring opening with NaOH followed by Ca^2+^ coordination crosslinking	Water	~48 h	Rose Bengal	>98	Support layer	[47]
P84 PI	Activation of PI with Tris and then crosslinking with Diamine (HDA)	Isopropyl alcohol	24 h	PS (200)	∼93	OSN	[48]
P84 PI	Crosslinking with diamine (HDA)	Ethanol	24 h.	RB (974)	97.74	Substrate	[49]
P84 PI	Thermal annealing at 330 °C followed by solvent activation	DMF (Solvent activation)	~ 4 h + 12 h (Solvent activation)	Food yellow 3 (452.36Da)	99.0	OSN	[50]
Synthesized PI (TMPDA + DDM + PMDA)	Crosslinking with MPD	Water	24 h	Salt rejections	~97.45	Substrate	[51]
Lenzing P84 PI	Crosslinking with diamine (HDA)	Isopropyl alcohol, DMF (Solvent activation)	1 h + (30 min (Solvent activation)	Rhodamine B	98.6%	Substrate	[52]
Lenzing P84 PI	Crosslinking with diamine (HDA)	Isopropyl alcohol, DMF (Solvent activation) alcohol, Hexane	1 h + (30 min (Solvent activation)	Rhodamine B	>99%	Substrate	[53]

## Data Availability

Not applicable.
